# A Population Model of Time-Dependent Changes in Serum Creatinine in (Near)term Neonates with Hypoxic-Ischemic Encephalopathy During and After Therapeutic Hypothermia

**DOI:** 10.1208/s12248-023-00851-0

**Published:** 2023-12-05

**Authors:** Wojciech Krzyzanski, Pia Wintermark, Pieter Annaert, Floris Groenendaal, Suzan Şahin, Mehmet Yekta Öncel, Didem Armangil, Esin Koc, Malcolm R. Battin, Alistair J. Gunn, Adam Frymoyer, Valerie Y.-L. Chock, Elif Keles, Djalila Mekahli, John van den Anker, Anne Smits, Karel Allegaert

**Affiliations:** 1 Department of Pharmaceutical Sciences, University at Buffalo, 370 Pharmacy Building, Buffalo, New York 14214, USA; 2 Division of Newborn Medicine, Department of Pediatrics, McGill University, Montreal Children’s Hospital, Research Institute of the McGill University Health Centre, Montreal, Quebec, Canada; 3 Department of Pharmaceutical and Pharmacological Sciences, KU Leuven, Louvain, Belgium; 4 Department of Neonatology, Wilhelmina Children’s Hospital, University Medical Center Utrecht, and Utrecht University, Utrecht, The Netherlands; 5 Utrecht Brain Center, University Medical Center Utrecht, Utrecht, The Netherlands; 6 Department of Neonatology, Faculty of Medicine, Izmir Demokrasi University, Izmir, Turkey; 7 Department of Neonatology, Faculty of Medicine, İzmir Katip Çelebi University, İzmir, Turkey; 8 Neonatal Intensive Care Unit, Koru Hospital, Ankara, Turkey; 9 Department of Neonatology, Faculty of Medicine, Gazi University, Ankara, Turkey; 10 Newborn Service, Auckland District Health Board, Auckland, New Zealand; 11 Department of Physiology, University of Auckland, Auckland, New Zealand; 12 Neonatal and Developmental Medicine, Stanford University School of Medicine, Palo Alto, California, USA; 13 Department of Pediatric Nephrology, University Hospitals, Louvain, Belgium; 14 PKD Research Group, Department of Cellular and Molecular Medicine, KU Leuven, Louvain, Belgium; 15 Division of Clinical Pharmacology, Children’s National Hospital, Washington, District of Columbia, USA; 16 Division of Paediatric Pharmacology and Pharmacometrics, University Children’s Hospital Basel, University of Basel, Basel, Switzerland; 17 Department of Development and Regeneration, KU Leuven, Louvain, Belgium; 18 Neonatal Intensive Care Unit, University Hospitals Leuven, Louvain, Belgium; 19 Department of Pharmaceutical and Pharmacological Sciences, KU Leuven, 3000 Louvain, Belgium; 20 Department of Hospital Pharmacy, Erasmus MC University Medical Center, 3015 Rotterdam, The Netherlands

**Keywords:** acute kidney injury, hypoxic-ischemic encephalopathy, neonates, population model, serum creatinine, therapeutic hypothermia, time-dependent covariate

## Abstract

The objective was to apply a population model to describe the time course and variability of serum creatinine (sCr) in (near) term neonates with moderate to severe encephalopathy during and after therapeutic hypothermia (TH). The data consisted of sCr observations up to 10 days of postnatal age in neonates who underwent TH during the first 3 days after birth. Available covariates were birth weight (BWT), gestational age (GA), survival, and acute kidney injury (AKI). A previously published population model of sCr kinetics in neonates served as the base model. This model predicted not only sCr but also the glomerular filtration rate normalized by its value at birth (GFR/GFR_0_). The model was used to compare the TH neonates with a reference full term non-asphyxiated population of neonates. The estimates of the model parameters had good precision and showed high between subject variability. AKI influenced most of the estimated parameters denoting a strong impact on sCr kinetics and GFR. BWT and GA were not significant covariates. TH transiently increased _sCr_ in TH neonates over the first days compared to the reference group. Asphyxia impacted not only GFR, but also the sCr synthesis rate. We also observed that AKI neonates exhibit a delayed onset of postnatal GFR increase and have a higher sCr synthesis rate compared to no-AKI patients. Our findings show that the use of sCr as marker of renal function in asphyxiated neonates treated with TH to guide dose selection for renally cleared drugs is challenging, while we captured the postnatal sCr patterns in this specific population.

## Introduction

Perinatal asphyxia is a condition at delivery, caused by oxygen and/or perfusion deprivation long enough to potentially result in sequelae, such as hypoxic-ischemic encephalopathy (HIE) or cerebral palsy (1). In neonates with moderate to severe HIE, therapeutic hypothermia (TH) is an effective intervention to significantly reduce mortality and morbidity (2). However, asphyxia is a multi-organ disease that is not limited to the central nervous system (3). As vaso-reactive organs, the kidneys are very sensitive to oxygen deprivation, frequently (39–42%) resulting in acute kidney injury (AKI) as part of a “perinatal asphyxia syndrome” (4). Interestingly, TH is also reno-protective if measured by the incidence of AKI (5). Several studies have quantified the impact of asphyxia and TH on the glomerular filtration rate (GFR) and GFR related drug clearance. Renal elimination was reduced (from −25 to −40%), or even up to −60% when based on mannitol clearance (6, 7). Except for one dataset on gentamicin clearance, most data have focused on the TH time window (the first 3 days of life), with much less information beyond this TH time window. A systematic literature search approach found that changes in creatinine in this specific subpopulation were inconclusive during the remainder of the first week of postnatal life (8).

Different modeling approaches have been used to quantify the impact of TH on GFR and renally cleared drugs. Favié *et al*. (9) used body temperature as a time dependent variable affecting drug clearance in a population pharmacokinetic (PK) model that estimated typical values of clearance of several drugs in critically ill neonates with HIE. Others proposed a physiologically based PK (PBPK) framework to describe drug concentrations in the organs and tissues of the neonate, where TH was used as a categorical covariate indicating changes of physiological parameters affected by hypothermia (10). While a TH-PBPK is still under development, TH was used as a dichotomous covariate affecting clearance in population PK models of plasma drug concentrations in asphyxiated neonates (7,11). Such a TH-PBPK development takes time, as such a workflow should be driven by integration of *in vivo* time-dependent pathophysiological (renal, cardiac, metabolism) patterns, real world data in this specific population, combined with *in vitro* data (e.g., drug metabolism) to quantify the impact of hypothermia in addition to the asphyxia and compared to reference data. Such reference data are already present in PBPK models (10).

We recently reported a mechanistic population model for serum creatinine (sCr) as time dependent (age, gestational, and postnatal) covariate in neonates (12). This is of relevance as sCr is commonly used as covariate in population pharmacokinetic models of drugs in neonates as a surrogate marker for GFR. The absence of time dependent covariates is a particular challenge for population modeling of such pharmacokinetic data. The objective of this analysis was to build on this model to describe the time course and variability of sCr in a subgroup of (near)term neonates with moderate to severe HIE during and after TH compared to a reference population. Our intention is that such model—weighted in a time-dependent way to a reference population—can be used as part of future full random effect models over time of renally cleared drugs in this specific population, capturing the relevant time window during and after TH (8).

## Methods

### Data

The data used for analysis have been previously published (13). Briefly, the data consisted of sCr observations in (near)term neonates with moderate-to-severe HIE, who underwent TH during the first 3 days after birth. The data covered the first 10 days of life. The available covariates were birth weight (BWT), gestational age (GA), survival (DEATH, neonatal death, day 1–28), and acute kidney injury (AKI). AKI detection was based on the KDIGO definition (any AKI): sCr↑≥0.3mg/dL within 48 h or sCr↑≥1.5 fold *versus* the lowest prior sCr within 7 days, irrespective of urine output, as these data were unavailable (13). Information on the severity (moderate or severe) is not available in the pooled sCr dataset. The reference population comprised of neonates with a GA of at least 24 weeks with at least one measurement of sCr during 42 days after birth and sCr value ≤ 2 mg/dL who were included in the model development dataset of (12). The reference dataset consisted of 1080 subjects with 7977 sCr observations. The descriptive statistics of covariates for TH and reference populations are presented in Table I. The sCr values obtained by the Jaffe assay were converted to values equivalent to ones obtained by an isotope dilution mass spectrometry (IDMS) traceable enzymatic assay using the following Eq. (1):

(1) $${sCr}_{\text{IDMS}}=1.003•{sCr}_{Jaffe}+0.057$$

**Fig. 1:**
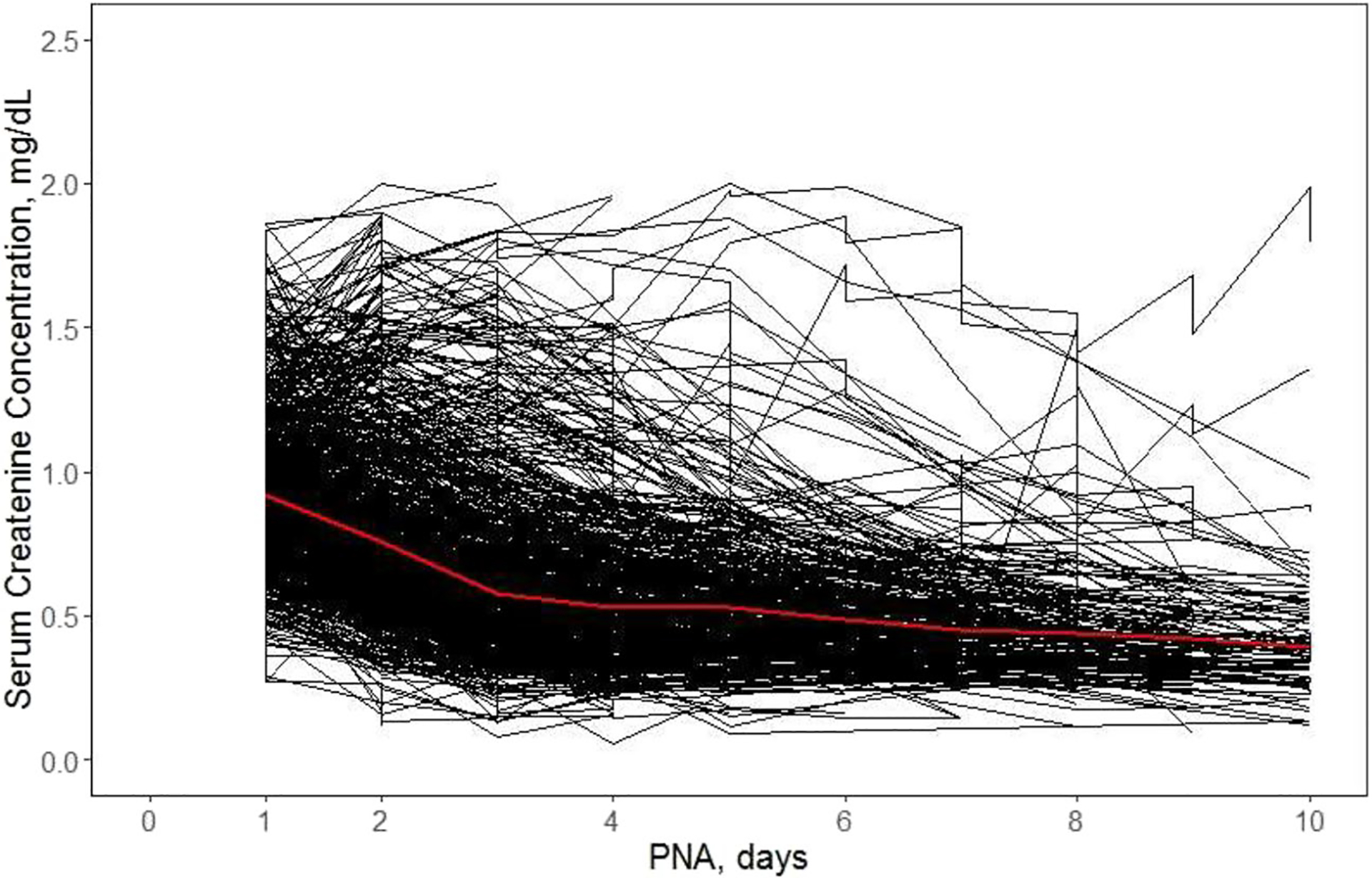
Time courses of individual serum creatinine (sCr) measurements for 975 in (near) term neonates with moderateto-severe hypoxic-ischemic encephalopathy who underwent therapeutic hypothermia (TH) during the first 3 days of life. The bold (red) line is the mean of observed values

The sCr values of obtained by an unidentified assay were excluded from the analysis. To facilitate application of a population model that was developed for the reference population, the data were further reduced to include subjects with sCr value ≤ 2 mg/dL and GA ≤ 42 weeks not exceeding the reference ranges. The final dataset consisted of 975 subjects with 4636 sCr observations (initial dataset had 1136 subjects and 4724 sCr observations). A spaghetti plot of individual sCr time courses is shown in Fig. 1. SCr data were reported in mg/dL (conversion, 1mg/dL = 88.4 μmol/L). A histogram of the GA distribution is shown in Fig. 1S.

#### Population Kinetic Model for Serum Creatinine

**Fig. 2:**
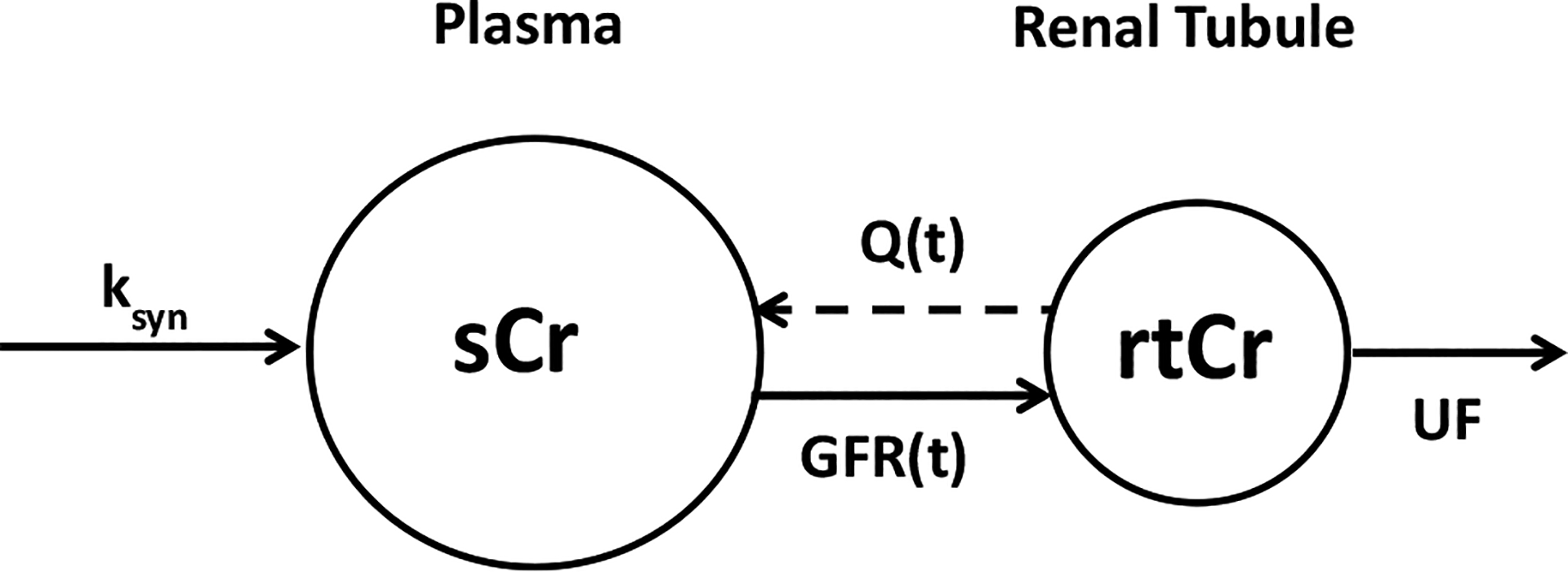
Schematic diagram of pharmacokinetic model of creatinine. Serum creatinine (sCr) is synthesized from the muscle at a zero-order rate $\left(k_{\text{syn}}\right)$ and cleared by the kidney glomerular filtration (GFR(t)) into the renal tubules (rtCr). The creatinine from the renal tubules is excreted into the urine by the urinary flow (UF). Transiently, rtCr is also secreted into the plasma by the back-flow (Q(t))

We adopted our previously introduced population model of sCr kinetics in neonates (see Fig. 2) (12):

(2) $$sCr=\left(1+\frac{Q(PNA)}{UF}\right)\frac{k_{syn}}{GFR(PNA)}$$

where $k_{\text{syn}}$ is the rate sCr is secreted from the muscle, Q(PNA) is a back-flow of creatinine from the renal tubules to the plasma, and GFR(PNA) is the glomerular filtration rate at the postnatal age PNA.Q(PNA) was modeled by the truncated Gaussian distribution function designed to describe peak and a gradual disappearance of the back-flow:

(3) $$Q(PNA)=\frac{Q_{max}}{UF}\cdot exp\;\left(-\frac{K^{2}}{2}\cdot {\left(PNA-{PNA}_{p}\right)}^{2}\right)$$

where $Q_{max}$ is the peak of the back-flow that occurs at time PNA_p_. The parameter K is the time scale factor that controls the width of Q(1/Kis equal to the standard deviation of the Gaussian distribution). GFR(PNA) was described by the sigmoidal $E_{max}$ model:

(4) $$GFR(PNA)={GFR}_{0}+\frac{\left({GFR}_{ss}-{GFR}_{0}\right)\cdot {PNA}^{\gamma }}{{PNA}_{50}^{\gamma }+{PNA}^{\gamma }}$$

where GFR_0_ is the GFR at birth, PNA_50_ is the time after birth at which 50% of GFR_ss_ is reached, and γ is the Hill coefficient.

All identifiable model parameters (P) were assumed to be log-normally distributed among subjects:

(5) $$P=\theta_{P}exp\;\left(\eta_{P}\right)\;\text{and}\;\eta_{\text{P}}∼N\left(0,\omega_{\text{P}}^{2}\right)$$

where $P\in \left\{Q_{max}/UF,{PNA}_{p},K,{GFR}_{ss}/{GFR}_{0},k_{syn}/{GFR}_{0},{PNA}_{50},\gamma \right\}$. The GA was identified as the only significant covariate (alternative to the body weight) and influencing the model parameters $P\in \left\{Q_{\text{max}}/{UPNA}_{p},K,{GFR}_{ss}/{GFR}_{0},k_{syn}/{GFR}_{0},{PNA}_{50},\right\}$ through the linear relationship

(6) $$P=\theta_{P}\left(1+\theta_{\text{COV\_P}}\left(GA-{GA}_{\text{mean}}\right)\right)exp\;\left(\eta_{P}\right)$$

where ${GA}_{\text{mean}}=34.2$ weeks.

The additional covariates in addition to GA for the current analysis were acute kidney injury (AKI) and patient’s death, defined as neonatal death (day 1–28) (DEATH). Both covariates were dichotomous with the value 1 indicating presence and 0 absence of the covariate:

(7) $$\theta_{P}=\theta_{P,COV=1}•COV+\theta_{P,COV=0}•(1-COV)$$

The constant residual error model was applied to the log-transformed sCr data:

(8) $$log\;\left({sCr}_{ij}\right)=log\;\left(sCr\left({PNA}_{j}\right)\right)+\varepsilon_{ij}\;\text{and}\;\varepsilon_{\text{ij}}\sim N\left(0,\sigma^{2}\right)$$

where ${sCr}_{ij}$ are sCr observations for subject i at age ${PNA}_{j}$.

#### Evaluation of Model Performance

The performance of the population model was evaluated using the diagnostic plots observed *vs*. predicted values, conditional individual weighted residuals (CWRES), conditional individual weighted residuals (CIWRES), and visual predictive check (VPC). To obtain VPC, 500 simulations of the data were performed with parameter estimates from the final model. Simulated 5th, median, and 95th percentiles and their 95% CIs were compared with observed values. The plots were generated in RStudio.

#### Simulations

Individual time courses of sCr and GFR/GFR_0_ for TH and a reference neonate (GA = 39 weeks, AKI = 0, and DEATH = 0) were simulated using estimated model parameters for both populations. For each population, 700 individual time courses with no residual error were generated at PNA increments 0.1 day over the PNA range of 10 days. At each PNA, the median, 5th, and 95th percentiles of sCr and GFR/GFR_0_ were calculated and plotted against PNA. Additionally, for TH neonates, analogous plots were generated of GA = 39 weeks, AKI = 1, and DEATH = 0.

#### Software

All models were implemented in NONMEM 7.4 (ICON Development Solutions, Ellicott City, MD). Population model parameters were estimated using the importance sampling (IMP) method with CTYPE = 1 convergence criterion. The model performance diagnostic plots were obtained using R packages: ggplot2, lattice, and vpc (14) implemented in RStudio version 2022.07.2 (RStudio Inc., Boston, MA).

## Results

The population analysis of sCr kinetics in TH patients consisted of estimation of model parameters, selection of significant covariates, and evaluation of the final model performance. The comparison of sCr and GFR values between TH and the reference population was done by simulating the time courses of these variables predicted by the models developed for both populations.

### Observed Serum Creatinine in the TH Cohort

Individual sCr time courses are shown in Fig. 1. The data exhibit high between subject variability. The mean sCr at PNA day 1 of about 0.92 mg/dL declines to 0.57 mg/dL by day 3 and continues to decrease at a much slower rate to reach the value of 0.39 mg/dL on day 10. One can observe a small peak on day 5.

### Estimation of Model Parameters

The population model Eqs. (1)–(4) was fitted to the log-transformed sCr data for the TH patients. Since the backflow of creatinine from the renal tubules to the plasma is minimal in neonates with GA>34 weeks (see Fig. 8 in (12)), the sCr peak parameters $Q_{max}/UF,K$, and PNA pere not identifiable. We therefore fixed their values as obtained for the reference population. The original covariate relationships between GA, $Q_{max}/UF$, and PNA_p_ were preserved. The typical and individual values for the remaining parameters ${PNA}_{50},{GFR}_{ss}/{GFR}_{0},k_{syn}/{GFR}_{0}$, and γ were estimated without any covariate relationships (Table II). Estimates of parameter typical values and their interindividual variability (IIV) were obtained with good precisions (%RSE less than 27%). A high between subject variability (greater than 65%) was observed for PNA_50_ and γ. The biggest difference in the estimates of the base model parameters for TH neonates and reference neonates of GA = 39 weeks was observed for PNA_50_ (2.31 *vs*. 18.2 days), $k_{\text{syn}}/{GFR}_{0}$ (0.654 *vs*. 0.394 mg/dL), and γ (5.26 *vs*. 3.57). GFR_ss_/GFR_0_ (1.78 *vs*. 2.01) differed by less than 13%.

### Covariate Analysis

Individual estimates of the base model parameters were correlated with two available covariates in addition to GA, AKI, and DEATH, respectively. The shrinkages of the distributions of individual parameter estimates were in the range 29.3–67.9%. The highest shrinkage was for γ. Only parameters with a correlation coefficient $r^{2}>0.1$ were added to the model to test for significance. Since GA poorly correlated with individual parameters, this covariate was excluded from further analysis. Similarly, AKI and DEATH poorly correlated with ${GFR}_{ss}/{GFR}_{0}\left(r^{2}<0.03\right)$, and this parameter was excluded from the log-likelihood ratio test. The forward-inclusion backward-elimination technique for covariate selection was applied (15). We used the log-likelihood ratio test of the change in the objection function value with significance levels for inclusion and elimination 0.001 and 0.0001, respectively. Table IS shows successful steps in the covariate selection process. AKI and DEATH significantly contributed to IIV of the following parameters $k_{\text{syn}}/{GFR}_{0},{PNA}_{50}$, and γ. The parameter estimates of the final population model including the covariate effects are presented in Table I. Table IIS compares final parameter estimates between reference and TH models.

### Evaluation of Model Performance

**Fig. 3:**
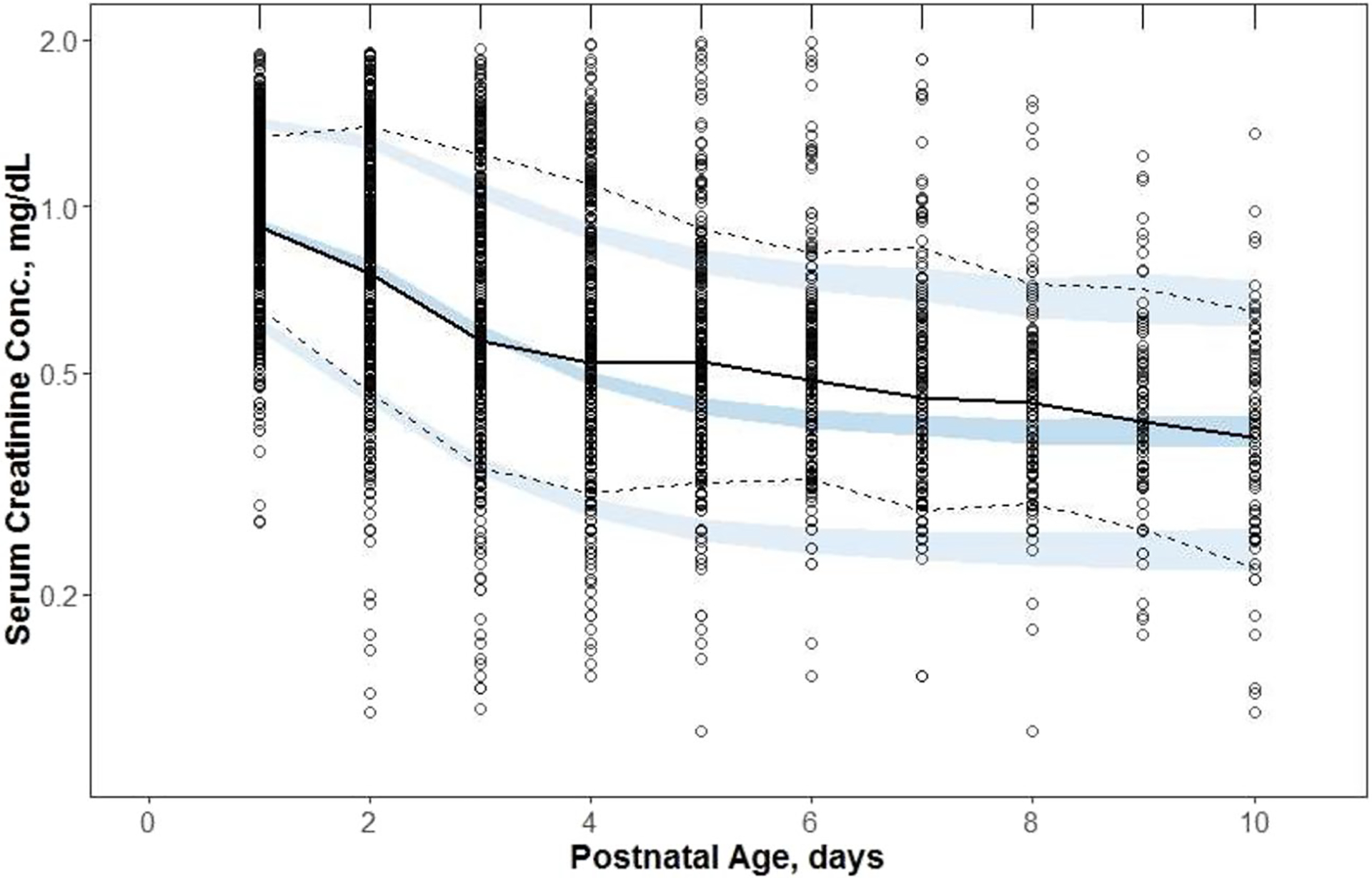
The visual predictive check plot for serum creatinine (sCr) from (near)term neonates who underwent therapeutic hypothermia (TH) because of moderate-to-severe hypoxic-ischemic encephalopathy. Symbols represent observed sCr, the continuous line is the median, and the dashed lines are 5th and 95th percentiles of observed values. The shaded regions are model predicted confidence intervals for these percentiles

Observed *vs*. predicted values, CWRES and CIWRES *vs*. PNA diagnostic plots are shown in Figs. 2S and 3S. While observed *vs*. predicted plots imply good agreement of model predicted sCr with the data, both CWRES and CIWRES plots indicate underprediction of sCr for PNA between 4 to 8 days. This is also observed in the vpc plot (see Fig. 3). The observed sCr transiently rise about PNA day 5; whereas, the model predicts a strict decrease during that time which causes the underprediction. The 5th and 95% percentiles of the model predicted sCr underpredicted analogous percentiles of the observed sCr for the same reasons. However, the difference between estimated variability and sCr data is not substantial, and the trend in data is captured by the model.

### Simulations of sCr and GFR Time Courses

**Fig. 4:**
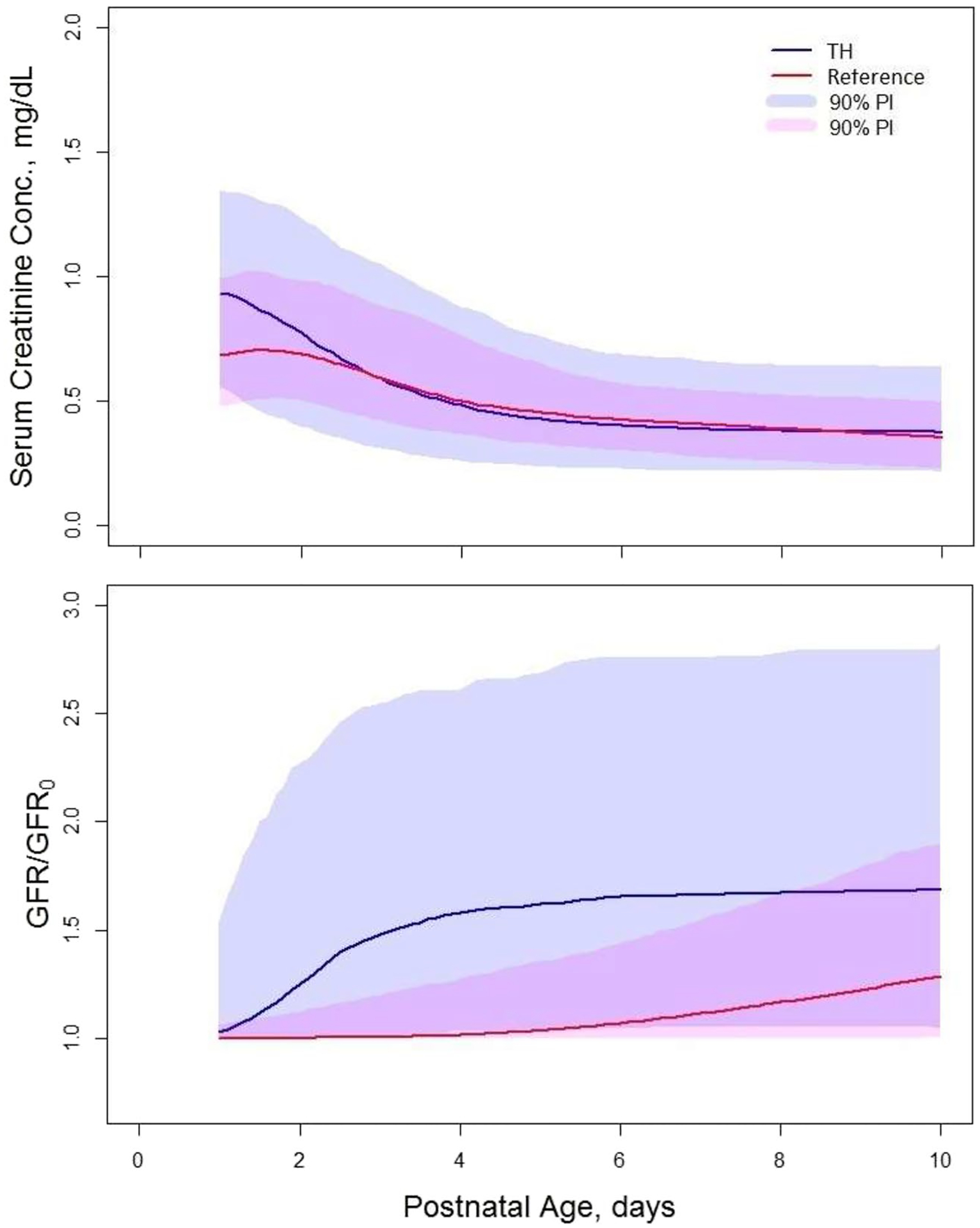
The median serum creatinine (sCr) *vs*. postnatal age (PNA) (upper panel) and glomerular filtration rate (GFR/GFR_0_) *vs*. PNA (lower panel) plots for therapeutic hypothermia (TH) neonates without acute kidney injury (AKI) who survived past day 28 of life, and reference neonates of gestational age (GA) = 39 weeks. The median values were calculated for simulated values for 700 neonates using the estimated values of final model parameters from Table II and Table I (12). The residual error was not included in the simulations. The shaded regions are 90% prediction intervals

The median sCr *vs*. PNA plots for TH neonates without AKI who survived past day 28 of treatment and reference neonates of GA = 39 weeks are shown in Fig. 4. The sCr values for TH neonates are higher than for the reference neonates up to PNA day 3, after which they become similar. The biggest difference between median sCr occurs on PNA = 1 day, and it is 0.24 mg/dL. The simulations reflect high variability in sCr between both populations as their 90% prediction intervals on the observed differences do not overlap statistically significant. The population model allowed simulation of time courses of median GFR/GFR_0_ as shown for both populations in Fig. 4. Both median GFR/GFR_0_ *vs*. PNA plots start at 1 and increase over the PNA range of 10 days. There is a relevant difference in their patterns. For the TH neonates, GFR/GFR_0_ rapidly increases from day 1 onwards, to reach a plateau of 1.65 on PNA day 6; whereas, GFR/GFR_0_ for reference neonates slowly increase to 1.3 without reaching a plateau on day 10. The difference between median GFR/GFR_0_ values on PNA is 0.4. Similarly to sCr, the 90% prediction intervals overlap rendering the differences statistically insignificant.

**Fig. 5:**
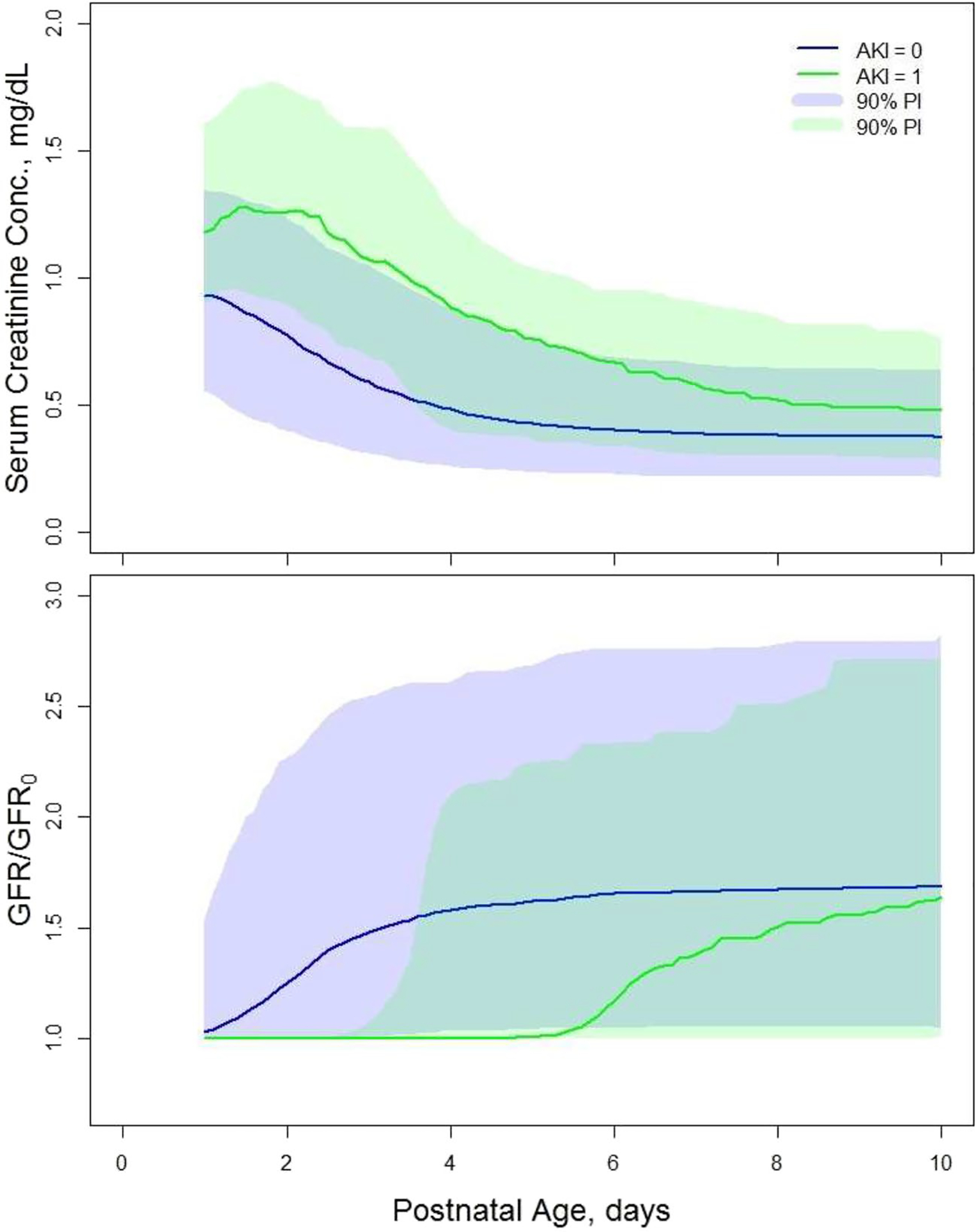
The median serum creatinine (sCr) vs. postnatal age (PNA) (upper panel) and glomerular filtration rate (GFR/GFR_0_) vs. PNA (lower panel) plots for therapeutic hypothermia (TH) neonates with or without acute kidney injury (AKI) who survived past day 28 of life, and with a gestational age of (GA) = 39 weeks. The medians were calculated for simulated values for 700 neonates using the estimated values of final model parameters from Table II and Table I (12). The residual error was not included in the simulations. The shaded regions are 90% prediction intervals

To determine the association between AKI and sCr and GFR in the TH neonates, we simulated time courses of the median sCr and GFR/GFR_0_ for neonates of GA = 39 weeks with and without AKI who survived past day 28 of life. The plots are shown in Fig. 5. The sCr values for neonates with AKI are higher than for neonates without AKI with the biggest difference of 0.5 mg/dL between PNA days 2–3. This difference diminishes over time to become 0.1 mg/dL on PNA day 10. The GFR/GFR_0_ values are lower for neonates with AKI than for neonates without AKI. In addition, the time course of GFR/GFR_0_ is markedly different for AKI patients. It remains at value of 1 up do PNA day 5, after which it rapidly increases towards the plateau for non-AKI patients on PNA day 10. The biggest difference 0.6 in GFR/GFR_0_ values occurs on PNA day 5.

## Discussion

In the present study, we used a population model initially developed and validated in a heterogenous population of neonates with no TH-related interventions, including preterm and term subjects. Blood samples to assess for sCr in this initial cohort were collected up to 42 days (12). This model allowed us to observe and quantify the processes absent in the currently studied dataset, such as back-flow of creatinine from the renal tubules to the plasma or reaching a steady-state by sCr. Consequently, we kept the part of the model related to the back-flow peak in sCr unchanged and used only the model components describing GFR and synthesis of creatinine. The current data involved a much smaller range of GA (34–42 weeks) compared to the reference dataset (25–42 weeks), because TH is only established for use in (near)term neonates. Both datasets exhibited high variability in the observed individual time courses of sCr that resulted in high values of the IIV parameter estimates and eliminated statistical significance of the between modelpredicted sCr and GFR values for the two populations, reflecting the clinical reality.

We estimated typical values, IIV, and individual values for four model parameters ${PNA}_{50},{GFR}_{ss}/{GFR}_{0},k_{syn}/{GFR}_{0}$, and γ. Except for GFR_ss_/GFR_0_, their estimates were more than 50% different from estimates on analogous parameters for the reference population. This denotes differences in GFR and creatinine synthesis in TH neonates that were manifested in different observed mean sCr time courses. $k_{\text{syn}}/{GFR}_{0}$ was 66% higher for the TH neonates, indicating a substantial increase in sCr production for this patient population most likely due to tissue injury, secondary to hypoxia–ischemia. Consistent with this hypothesis, Valerio *et al*. observed higher creatine (precursor of creatinine) urine elimination in neonates with HIE and suggested that this reflects enhanced creatine mobilization from the liver (16). ${GFR}_{ss}/{GFR}_{0}$ was only slightly lower (−13%) in TH neonates compared with the reference population, implying that their GFR reaches a broadly comparable state to the reference neonates. This reflects to a large extent clinical experience that renal function commonly normalizes by day 10 in most cases (13). The parameters PNA_50_ and γ control the shape of the GFR time course and variation in their values result in differences in GFR *vs*. PNA patterns between the two populations, confirmed by the simulations. The estimated IIV of these parameters converted to percent of coefficient variations were in the range 13–50%, consistent with the high variability in observed absolute sCr values.

The available covariates were body weight at birth, GA, AKI, and DEATH. Since GA rather than body weight was used in our reference model, we did not consider the latter for covariate analysis. The correlations between GA and individual estimates of model parameters were weak ($r^{2}<0.1$), and consequently, GA was not included in the covariate relationships. Such weak correlations can be explained by the relatively abbreviated range of GA (34 ≤ GA ≤ 42 weeks) in our study. AKI was the most influential covariate affecting all parameters, but GFR_ss_/GFR_0_. This implies that AKI transiently affects GFR in TH neonates and its steady state eventually is same as for the TH neonates without AKI. AKI increases the rate of sCr synthesis by 24% in the TH neonates who survived 28 days after the birth and 11% in the neonates who did not. This reflects the fact that the AKI definition and its severity are in part determined by the increase in sCr.

The applied diagnostics of model performance indicate some imperfections. The most notable one was an underprediction of sCr for PNA between 4 to 8 days. The mean sCr values in the TH neonates show a small peak at PNA = 5 days; whereas, such a peak is absent in the mean sCr time courses for the reference neonates. This feature of the data is likely attributed to asphyxia or TH, or is a bias related to the retrospective data collection, likely driven by clinical indications. It would require an additional component that is not present in our reference model used for data analysis. Another imperfection in model performance is the relatively narrow 95% CIs for model predicted 5th, 50th, and 95 th percentiles of sCr given the high variability of sCr values. This simply reflects the large number of subjects in our data set that contributes to the limits of 95% CI. None of these issues distorts model performance and prediction power. Overall, the model shows good agreement between observed and predicted values as well as it provides estimates of model parameters with good precisions.

The estimated model parameters were used to simulate the time courses of sCr and GFR/GFR in TH and reference neonates. We observed that the median sCr is higher for TH neonates up to PNA day 3 after which they become similar. This is consistent with the comparison of the median sCr based on the observed data reported by Keles *et al*. (13). The simulations of GFR/GFR_0_ *vs*. PNA plots revealed that GFR/GFR_0_ is not only higher for TH neonates but also it increases much faster in the initial days after birth than in the reference neonates. Favié *et al*. (9) introduced a “maturation function” to account for TH cases with higher maturation compared to controls. Since renal drug clearance in TH neonates is decreased compared to non-TH neonates (6, 7), one would expect lower GFR for the former than for the latter. This seemingly paradoxical behavior of GFR/GFR_0_ can be explained by the lower initial GFR values at birth (GFR_0_) for neonates treated with TH than for the reference neonates. The same pattern (lower at the start, with subsequent faster “catch-up” in TH cases) has been described for renally cleared drugs (9). The rapid increase in GFR/GFR_o_ between PNA 2–4 days coincides with the termination of TH after 3 days following the delivery. This could reflect either increasing clearance after rewarming or non-causal collinearity of the intervention with PNA.

AKI influenced most of the estimated parameters implying its strong impact on sCr and GFR. Our simulations of sCr *vs*. PNA and GFR/GFR_0_ *vs*. PNA plots in the TH neonates who survived 28 days after the birth revealed that sCr is higher for neonates with AKI than without it. This is consistent with the observed sCr values in TH neonates stratified by presence of AKI reported by Keles *et al*. (13). The model predicted GFR/GFR_0_ time course in the neonates with AKI is somewhat delayed by about 5 days compared to the neonates without AKI followed by a similar rapid increase. Both groups of patients have similar sCr and GFR/GFR_0_ at PNA day 10 that implies that AKI impacts the GFR mostly by delaying its postnatal onset of its expected increase. The increase in sCr during this lag time is likely caused by the 24% higher production rate of sCr for AKI neonates compared to non-AKI neonates (16).

Drug dosing in asphyxiated neonates treated with TH is challenging not only because of dynamic change of the body weight but also because of the impact of the disease severity and TH on the renal function. Comparing GFR between treated and reference patients is a first step towards dosing adjustment for renally cleared drug. Population PK models for renally eliminated drugs administered to this specific subpopulation have been developed (9, 11). Body weight and GA are commonly used as explanatory covariates for quantification of clearance variability. Inclusion of sCr as a covariate would greatly enhance the model predictive power of the drug’s renal clearance in individual patients and further reduced the variability. Guidelines on including sCr as a covariate in population PK models for pediatric patients have been introduced (17). Population PK model-based guidelines of vancomycin dosing in neonates have been developed where sCr was successfully implemented as covariate (18). Our model should be useful to facilitate such implementations in population PK models of TH neonates. Furthermore, such time-dependent function is useful to predict renal drug clearance for trial design, or to quantify the impact of reno-protective interventions (like methylxanthines) in this specific population (19). It is hereby recognized that creatinine is likely not the most accurate biomarker of GFR or AKI. However, it is the most commonly collected biomarker in clinical care, and the Schwartz equation coefficient has recently been validated to measure GFR values in term neonates (20).

## Conclusion

The current population model of sCr kinetics in asphyxiated neonates treated with TH allowed us to quantify the available clinical data and compare these data to non-HIE, non-TH neonates with similar GA range. Additionally, we were able to infer the contributions of sCr synthesis and GFR to observed sCr time courses. Due to identifiability problems, model parameters and predicted GFR values were expressed relative to the GFR values at birth that somewhat impaired interpretation of the results. The TH transiently increases sCr in TH neonates compared to the reference group. We observed that asphyxia impacts not only GFR but also the sCr synthesis rate. Finally, we concluded that AKI neonates exhibit an additional delay in onset in the expected increase of GFR and have a higher SCr synthesis rate compared to no-AKI patients. Our findings highlight the challenges involved in using sCr as a marker of renal function in asphyxiated neonates treated with TH to guide dose adjustments for renally cleared drugs, while we captured the postnatal sCr patterns in this specific population.

## Supplementary Material

Supplementary_Material

## Figures and Tables

**Table I:** Descriptive Statistics of Available Covariates for Population of Neonates in Therapeutic Hypothermia (TH) and Reference Populations

Covariate	TH	Reference
Number of patients	975	1080
GA (weeks)	39 (34, 42)	35 (31, 38)
BWT (g)	3340 (1750, 6230)	2300 (1486, 3203)
PNA (day)	3 (1,10)	7 (3, 17)
AKI (0/1)	896/79	NA
DEATH (0/1)	827/148	NA

Continuous covariate values are presented as median (min, max) and discrete covariates as proportions

GA gestational age, BWT birth weight, PNA postnatal age, AKI, acute kidney injury, NA not applicable

**Table II:** Estimates and Their Percent Relative Standard Errors (%RSE) of the Population Parameters for the Base and Final Models

Parameter	Estimate of typical value (%RSE) Base	Estimate of IIV (%RSE, %CV) Base	Estimate of typical value (%RSE) Final	Estimate of IIV (%RSE, %CV) Final
PNA_50_(day)	2.31 (3.8)	0.413 (9.2, 71.5)	NA	0.220 (10.7, 49.6)
PNA_50AKI=0_(day)	NA	NA	1.94 (3.5)	NA
PNA_50AKI=1_(day)	NA	NA	6.21 (10.8)	NA
GFR_SS_/GFR_0_	1.78 (2.3)	0.107 (8.7, 33.6)	1.74 (2.0)	0.0955 (7.9, 31.7)
*k*_syn_/GFR_0_(mg/dL)	0.654 (1.4)	0.0233 (15.2, 15.4)	NA	0.0180 (16.6, 13.5)
*k*_syn_/GFR_0AKI=0DEATH=0_, (mg/dL	NA	NA	0.631 (1.4)	NA
*k*_syn_/GFR_0AKI=1DEATH=0_(mg/dL)	NA	NA	0.785 (2.9)	NA
*k*_syn_/GFR_0AKI=0DEATH=1_(mg/dL)	NA	NA	0.750 (2.9)	NA
*k*_syn_/GFR_0AKI=1DEATH=1_(mg/dL)	NA	NA	0.834 (4.4)	NA
*γ*	5.26 (9.2)	0.435 (18.8, 73.9)	NA	0.168 (28.4, 42.8)
*γ*_AKI=0_	NA	N A	4.46 (7.7)	NA
*γ*_AKI=1_	NA	NA	22.8 (28.2)	NA
*K*(1/day)	0.709** (3.3)	0.207** (13.9, 48.0)	0.709** (3.3)	0.207** (13.9, 48.0)
PNA_p_(day)	2.42** (1.5)	0.11** (7.0, 34.1)	2.42** (1.5)	0.11** (7.0, 34.1)
Q_max_/UF	0.575** (3.2)	0.153** (9.4, 40.7)	0.575** (3.2)	0.153** (9.4, 40.7)
GA_PNA_p_(day/week)	−0.0747** (4.8)	NA	−0.0747** (4.8)	NA
GA_Q_max_/UF(1/week)	0.0178** (27.9)	NA	0.0178** (27.9)	NA
σ^2^	0.0218 (3.3)	NA	0.0227 (3.2)	NA

Estimates of IIV parameters are presented as variance and coefficient of variation for lognormal distribution (%CV*)

NA not applicable,

$GFR_{ss}$ glomerular filtration rate (GFR) value at steady state, GFR GFR value at birth, $PNA_{50}$ postnatal age (PNA) at which GFR is equal to 50% of ${GFR}_{ss},k_{syn}$ sCr synthesis rate constant, γ Hill factor, K time scale factor that controls the width of the back-flow curve $Q,PNA_{p}$ PNA at the back-flow peak, $Q_{max}$ back-flow peak value, UF urinary flow, $GA_{\_}PNA_{p}$ slope in the covariate relationship between PNA_p_ and GA, $GA\_Q_{max}/UF$ slope in the covariate relationship between Q_max_/UF and $GA,\sigma^{2}$ variance of residual error for sCr observations

* $\%CV=100\%\sqrt{exp\;\left(\omega_{P}^{2}\right)-1}$

** Parameter was fixed at value obtained from (12)

## Data Availability

We encourage researchers to contact the corresponding author if there were interest in the data described. Any availability decision will be based on a protocol, while the individual centers remain the primary owners of the data.
