# Supplementary material for: A Population Model of Time-Dependent Changes in Serum Creatinine in (Near)term Neonates with Hypoxic-Ischemic Encephalopathy During and After Therapeutic Hypothermia
Source: AAPS J. Author manuscript; Available in PMC 2024 Jun 14. (PMC11177850; doi:10.1208/s12248-023-00851-0)
Supplement: Supplementary_Material [file NIHMS1999765-supplement-Supplementary_Material.docx]

**Supplementary Material**

A population model of time-dependent changes in serum creatinine in (near)term neonates with hypoxic-ischemic encephalopathy during and after therapeutic hypothermia**.**

Wojciech Krzyzanski, Pia Wintermark, Pieter Annaert, Floris Groenendaal, Suzan Şahin, Mehmet Yekta Öncel, Didem Armangil, Esin Koc, Malcolm R Battin, Alistair J. Gunn, Adam Frymoyer, Valerie Y-L Chock, Elif Keles, Djalila Mekahli, John van den Anker, Anne Smits, Karel Allegaert.

**Table 1S.** Objective function value (OFV) and its change (ΔOFV) for the successful steps of the forward-inclusion covariate selection process and the final model in the backward elimination process. P value is calculated for the chi square distribution of |ΔOFV| with one degree of freedom.

| Covariate relationship | OFV (ΔOFV) | P |
| --- | --- | --- |
| Base model | -7674.7 (0) | NA |
| $AKI\_\mathrm{PNA}_{50}$ | -7832.6 (-157.9) | 3.24E-36 |
| $AKI\_\mathrm{PNA}_{50}$ & AKI$\_k_{\mathrm{syn}}/\mathrm{GFR}_{0}$ | -7906.5 (-74.0) | 7.91E-18 |
| $AKI\_\mathrm{PNA}_{50}$ & AKI$\_k_{\mathrm{syn}}/\mathrm{GFR}_{0}$& AKI$\_\gamma$ | -7957.1 (-50.2) | 1.17E-12 |
| $\mathbf{AKI}\boldsymbol{\_}\mathbf{PNA}_{\mathbf{50}}$ **& AKI**$\boldsymbol{\_}\mathbf{k}_{\mathbf{syn}}\mathbf{/}\mathbf{GFR}_{\mathbf{0}}$**& AKI**$\boldsymbol{\_}\boldsymbol{\gamma}$**& DEATH**$\boldsymbol{\_}\mathbf{k}_{\mathbf{syn}}\mathbf{/}\mathbf{GFR}_{\mathbf{0}}$ | **-7996.9 (-90.3)** | **2.42E-20** |
| AKI$\_k_{\mathrm{syn}}/\mathrm{GFR}_{0}$ & AKI$\_\gamma$& DEATH$\_k_{\mathrm{syn}}/\mathrm{GFR}_{0}$ | -7857.01 (139.9) | 2.78E-32 |
| $AKI\_\mathrm{PNA}_{50}$ & AKI$\_\gamma$& DEATH$\_k_{\mathrm{syn}}/\mathrm{GFR}_{0}$ | -7963.2 (33.7) | 6.41E-09 |
| $AKI\_\mathrm{PNA}_{50}$ & AKI$\_k_{\mathrm{syn}}/\mathrm{GFR}_{0}$& DEATH$\_k_{\mathrm{syn}}/\mathrm{GFR}_{0}$ | -7942.7 (54.2) | 1.81E-13 |
| $AKI\_\mathrm{PNA}_{50}$ & AKI$\_k_{\mathrm{syn}}/\mathrm{GFR}_{0}$& AKI$\_\gamma$ | -7957.1 (39.8) | 2.27E-09 |

NA = not applicable

**Table 2S.** Estimates and their percent relative standard errors (%RSE) of the population parameters for the final reference and TH models. Estimates of IIV parameters are presented as variance and coefficient of variation for lognormal distribution (%CV*).

| Parameter | Estimate of typical value (%RSE)  Reference | Estimate of IIV  (%RSE, %CV)  Reference | Estimate of typical value (%RSE)  TH | Estimate of IIV (%RSE, %CV)  TH |
| --- | --- | --- | --- | --- |
| $\mathrm{PNA}_{50}$, day  ${GA\_PNA}_{50}$, day/week | 18.5 (3.0)  -0.0547 (16.9) | 0.145 (12.2, 39.5)  NA | NA  NA | 0.220 (10.7, 49.6)  NA |
| $\mathrm{PNA}_{50AKI=0}$, day | NA | NA | 1.94 (3.5) | NA |
| $\mathrm{PNA}_{50AKI=1}$, day | NA | NA | 6.21 (10.8) | NA |
| $\mathrm{GFR}_{\mathrm{ss}}/\mathrm{GFR}_{0}$  ${GA\_GFR}_{\mathrm{ss}}/\mathrm{GFR}_{0}$ | 1.97 (1.8)  0.00763 (30.9) | 0.0141 (18.9, 11.9)  NA | 1.74 (2.0)  NA | 0.0955 (7.9, 31.7)  NA |
| $k_{\mathrm{syn}}/\mathrm{GFR}_{0}$, mg/dL  ${GA\_k}_{\mathrm{syn}}/\mathrm{GFR}_{0}$, mg/dL/week | 0.516 (1.2)  -0.0255 (7.9) | 0.0169 (8.5, 13.1)  NA | NA  NA | 0.0180 (16.6, 13.5)  NA |
| $k_{\mathrm{syn}}/\mathrm{GFR}_{0AKI=0DEATH=0}$, mg/dL | NA | NA | 0.631 (1.4) | NA |
| $k_{\mathrm{syn}}/\mathrm{GFR}_{0AKI=1DEATH=0}$, mg/dL | NA | NA | 0.785 (2.9) | NA |
| $k_{\mathrm{syn}}/\mathrm{GFR}_{0AKI=0DEATH=1}$, mg/dL | NA | NA | 0.750 (2.9) | NA |
| $k_{\mathrm{syn}}/\mathrm{GFR}_{0AKI=1DEATH=1}$, mg/dL | NA | NA | 0.834 (4.4) | NA |
| $\gamma$ | 3.57 (5.3) | 0.52 (11.8, 82.6) | NA | 0.168 (28.4, 42.8) |
| $\gamma_{AKI=0}$ | NA | N A | 4.46 (7.7) | NA |
| $\gamma_{AKI=1}$ | NA | NA | 22.8 (28.2) | NA |
| $K$, 1/day | 0.709** (3.3) | 0.207** (13.9, 48.0) | 0.709** (3.3) | 0.207** (13.9, 48.0) |
| $\mathrm{PNA}_{p}$, day | 2.42** (1.5) | 0.11** (7.0, 34.1) | 2.42** (1.5) | 0.11** (7.0, 34.1) |
| $Q_{\max}/UF$ | 0.575** (3.2) | 0.153** (9.4, 40.7) | 0.575** (3.2) | 0.153** (9.4, 40.7) |
| $GA\_\mathrm{PNA}_{p}$, day/week | -0.0747** (4.8) | NA | -0.0747** (4.8) | NA |
| $GA\_Q_{\max}/UF$ , 1/week | 0.0178** (27.9) | NA | 0.0178** (27.9) | NA |
| $\sigma^{2}$ | 0.0151 (2.2) | NA | 0.0227 (3.2) | NA |

* $\%CV=100\%\sqrt{\exp\left( \omega_{P}^{2} \right)-1}$

** Parameter was fixed at value obtained from (12)

**
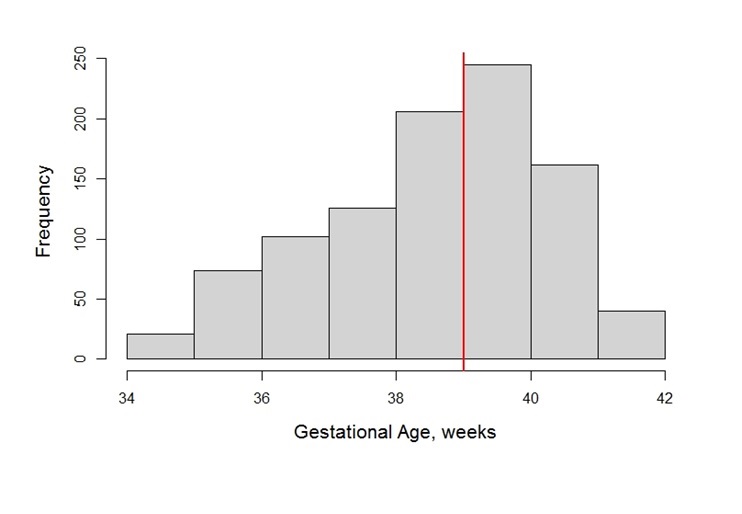
**

**Figure 1S.** The frequency histogram of gestational age for 975 in (near)term neonates who underwent whole body hypothermia during the first 3 days after the delivery because of moderate-to-severe hypoxic-ischemic encephalopathy. The bold line indicated the median gestational age of 39 weeks.

**
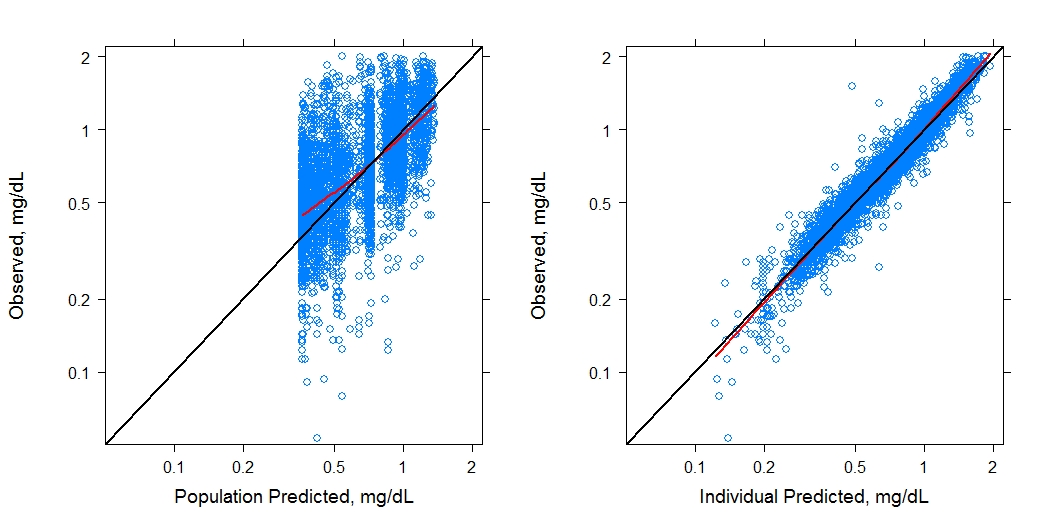
**

**Figure 2S.** Observed vs. Predicted diagnostic plots of the final model performance. The red lines are LOESS curves.

**
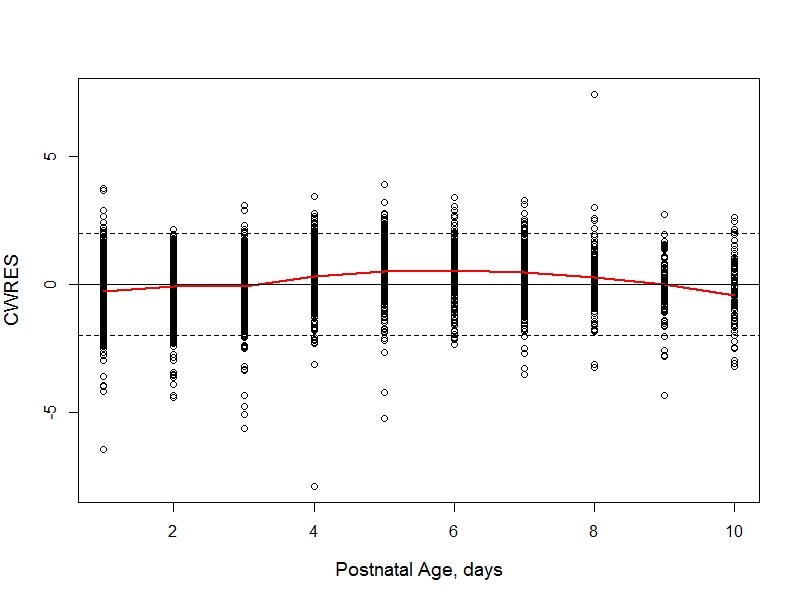
**

**
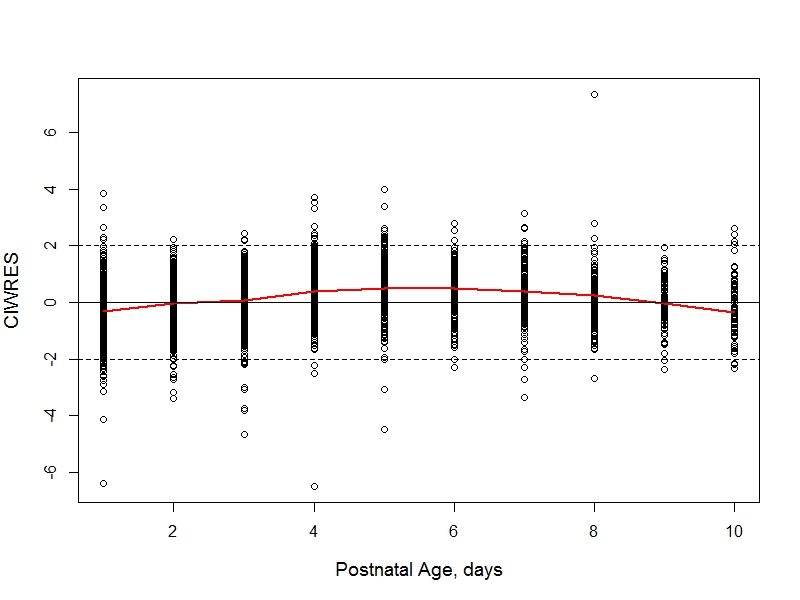
**

**Figure 3S.** Conditional weighted residuals (CWRES) vs. postnatal age (upper panel) and conditional individual weighted residuals (CIWRES) vs. postnatal age (lower panel) diagnostic plots of the final model performance. The red lines are LOESS curves.
